# Effects of the pattern of glucocorticoid replacement on neural processing, emotional reactivity and well-being in healthy male individuals: study protocol for a randomised controlled trial

**DOI:** 10.1186/s13063-016-1159-x

**Published:** 2016-01-22

**Authors:** Konstantinos Kalafatakis, Georgina M. Russell, Catherine J. Harmer, Marcus R. Munafo, Nicky Marchant, Aileen Wilson, Jonathan C. W. Brooks, Ngoc J. Thai, Stuart G. Ferguson, Kirsty Stevenson, Claire Durant, Kristin Schmidt, Stafford L. Lightman

**Affiliations:** Henry Wellcome Laboratories for Integrative Neuroscience and Endocrinology, School of Clinical Sciences, Faculty of Medicine and Dentistry, University of Bristol, Dorothy Hodgkin Building, Whitson Street, Bristol, BS1 3NY UK; Bristol Royal Infirmary, University Hospitals Bristol NHS Foundation Trust, Bristol, BS28HW UK; Clinical Research and Imaging Centre, University of Bristol, Bristol, BS28DX UK; Department of Psychiatry, Medical Sciences Division, University of Oxford, Oxford, OX37JX UK; MRC Integrative Epidemiology Unit at the University of Bristol, UK Centre for Tobacco and Alcohol Studies, School of Experimental Psychology, University of Bristol, Bristol, BS81TU UK; School of Medicine, University of Tasmania, Hobart, TAS 7000 Australia

**Keywords:** Glucocorticoid rhythmicity, Randomised controlled trial, Healthy volunteers, Functional neuroimaging, Emotional processing

## Abstract

**Background:**

Deviation from the physiological glucocorticoid dynamics (circadian and underlying ultradian rhythmicity) is a common characteristic of various neuropsychiatric and endocrine disorders as well as glucocorticoid-based therapeutics. These states may be accompanied by neuropsychiatric symptomatology, suggesting continuous dynamic glucocorticoid equilibrium is essential for brain homeostasis.

**Methods/design:**

The study consists of two parts. The preliminary stage of the study aims to validate (technically and pharmacologically) and optimise three different patterns of systemic cortisol administration in man. These patterns are based on the combinatory administration of metyrapone, to suppress endogenous cortisol production, and concurrent hydrocortisone replacement. The second, subsequent, core part of the study is a randomised, double-blinded, placebo-controlled, crossover study, where participants (healthy male individuals aged 18–60 years) will undergo all three hydrocortisone replacement schemes. During these infusion regimes, we plan a number of neurobehavioural tests and imaging of the brain to assess neural processing, emotional reactivity and perception, mood and self-perceived well-being. The psychological tests include: ecological momentary assessment, P1vital Oxford Emotional Test Battery and Emotional Potentiated Startle Test, Leeds Sleep Evaluation Questionnaire and the visual working memory task (n-back). The neuroimaging protocol combines magnetic resonance sequences that capture data related to the functional and perfusion status of the brain.

**Discussion:**

Results of this clinical trial are designed to evaluate the impact (with possible mechanistic insights) of different patterns of daily glucocorticoid dynamics on neural processing and reactivity related to emotional perception and mood. This evidence should contribute to the optimisation of the clinical application of glucocorticoid-based therapeutics.

**Trial registration:**

UK Clinical Research Network, IRAS Ref: 106181, UKCRN-ID-15236 (23 October 2013)

## Background

### Glucocorticoid dynamics and neurobehavioural equilibrium

The hypothalamic–pituitary–adrenal (HPA) axis is a complex homeostatic system, consisting of multiple feedforward and feedback regulatory interactions from the corticolimbic areas of the brain to the adrenal cortex [1]. These interactions result in both a circadian and an ultradian rhythm of cortisol secretion [2], which may be further modified by internal or external stressors [3].

HPA activity can be classified within two categories: (i) the regulation of internal homeostatic mechanisms (many of which have a circadian pattern) and (ii) the coordination of stress responses. Although there are glucocorticoid receptors in almost every tissue of the body, these hormones’ predominant effects relate to metabolic, immunological or cognitive functions. The wide spectrum of glucocorticoid-related biological actions has been exploited in the field of therapeutics for various disorders, though long-term use and/or high doses have been accompanied by numerous side effects [4].

Glucocorticoids modify the physiology of the central and peripheral nervous system at multiple levels. They interfere with the molecular processes involved in the modulation of the neuroinflammatory response, neurodegeneration and neuroregeneration/neurogenesis, neuronal viability and the cellular metabolic balance of neuronal and glial populations during developmental stages. Moreover, glucocorticoids regulate the state of activity of most systems of neurotransmission and synaptic plasticity, especially under acute stressful conditions or during chronic post-stress cognitive adaptations. Ultimately, glucocorticoids modify the status of emotional reactivity, cognitive precision and overall behaviour [1].

Glucocorticoid-dependent signalling has been associated with a number of neurological (stroke, traumatic brain injury, neurodegenerative disorders) and psychiatric disorders (depression, psychosis, post-traumatic stress disorders, antisocial behaviour, anxiety disorders and addiction). Similar problems, mainly fatigue, depression or dementia, seem to arise in patients whose treatment strategy involves high dose and/or long-term use of glucocorticoid-related therapeutics (even in cases of substitution therapy), as well as in patients with endogenous hypersecretion (Cushing’s syndrome) or deficiency (Addison’s disease, congenital adrenal hyperplasia) of glucocorticoids [4].

In all of these conditions there is a deviation from physiological glucocorticoid dynamics. The rationale for this protocol is therefore to examine whether glucocorticoid rhythmicity differentially regulates neural processing related to mood and emotional perception. These are cognitive aspects controlled via the corticolimbic areas of the brain and are susceptible to glucocorticoid effects.

### Aims of this paper

This article serves the following purposes: (1) the presentation of the study protocol and the underlying scientific evidence that led to its specific design, (2) the development of a metyrapone and hydrocortisone block and replace regime that allowed precise pharmacological manipulation of the underlying glucocorticoid rhythm, (3) the presentation of the temporal design of our magnetic resonance imaging (MRI) protocol and some technical aspects on approaching neuroimaging analysis, following the guidelines for reporting functional neuroimaging data.

### Rationale of the study

The importance of the glucocorticoid circadian rhythm in maintaining homeostasis has been long recognised. Nevertheless, there is little evidence available concerning the clinical impact of the underlying physiological ultradian (pulsatile) rhythm.

Preclinical research work over the last decade has highlighted the importance of ultradian pulses, which are synchronised between the blood, subcutaneous tissue and the brain [5], and lead to rapid and transient activation of its target receptors, resulting in dynamic genomic, neuroendocrinological and behavioural changes [6]. Moreover, they differentially affect neuronal plasticity and neurotransmission [7].

A number of clinical trials in healthy subjects, using functional neuroimaging techniques, have added valuable insights into the influence that glucocorticoids exert on cognitive and behavioural functions. Under such experimental settings, it has been shown that hydrocortisone administration imposes changes in emotional processing, attention or working memory related to either alterations in corticolimbic activity or differential functional connectivity [8–10]. Nevertheless, none of these clinical trials investigated the relevance of pattern administration (that is, the relevance of altered glucocorticoid rhythmicity) to these neurobehavioural changes. The focus was to contrast the state of high, stress-related glucocorticoid concentrations to the normal, non-stress levels, and study either the rapid (mediated by the activation of the membrane-associated glucocorticoid receptors) and/or the delayed (mediated by the activation of the nuclear glucocorticoid receptors) glucocorticoid effects on cognitive performance and emotional perception.

### Main research questions and hypothesis

Our research team has adopted a novel approach, which enables us to infuse replacement steroids in either a physiological circadian rhythm with its underlying ultradian rhythm or as a constant infusion, which abolishes the ultradian component of the circadian rhythm. This will allow us to test our hypothesis that the pattern of systemic glucocorticoid fluctuations leads to differential neurobehavioural phenotypes and activation of brain areas related to mood and emotional processing.

## Methods

### Overview of the study design

This is a physiological, pilot study on healthy, adult male volunteers, who will undergo three different strategies of hydrocortisone replacement after their endogenous glucocorticoid production is pharmacologically suppressed in a safe and reversible manner. The study has two stages: a preliminary stage (stage 1), where the pharmacological interventions are optimised and biochemically validated (presented here). The second stage of the study (stage 2) will be a double-blinded, placebo-controlled, three-way crossover, neurobehavioural study, in which healthy male subjects randomly alternate the three different modes of hydrocortisone replacement. The effect of each condition is assessed using functional and perfusion imaging of the brain and a series of psychological measurements assessing emotional reactivity and self-perceived well-being. For both study parts, the same regulations apply in terms of inclusion and exclusion criteria, the recruitment process and biomedical ethics. In the second stage of the study some additional inclusion and exclusion criteria, as well as steps in the recruitment process, will be applied (as discussed below).

### Inclusion and exclusion criteria

We shall recruit healthy volunteers, with no medical history and a normal physical examination assessed during the screening process by a qualified physician. Volunteers must not be casual smokers or consume excessive amounts of alcohol frequently, and additionally, they must not consume excessive amounts of caffeinated drinks (for details refer to Table 1). Since there are age-, gender- and working routine-specific factors that affect the mode of HPA axis function [11], this study is limited to male individuals, aged between 18–60 years old, who are not shift workers [12]. Furthermore, volunteers are excluded if they have participated in any other drug trial within the last two months prior to current study participation or have used any medication (prescribed, over the counter or recreational), including topical steroids and inhalers, within 48 hours of the current study initiation.

**Table 1:** Exclusion criteria of the study

1. Non-healthy individuals
• Previous medical history for any chronic condition in the last three months, active disease, or abnormal physical examination as verified by a qualified physician.
2. Casual smoking
• >6 cigarettes per day.
3. Frequent, heavy alcohol consumption
• >21 units/week.
4. Frequent, heavy caffeine consumption
• >4 caffeinated drinks/day.
5. Gender: female
6. Age: < 18 and > 60 years old
7. Shift workers
8. Participation in an investigational drug trial within the past two months
9. Intake of any medication (prescribed, over the counter or recreational) including topical steroids and inhalers, within 48 hours of the study initiation
• Urine sampling for testing for drugs of abuse^a^
Stage 1 (pharmacological validation) only
10. Needle phobia
Stage 2 (neurobehavioural investigation) only
11. Left-handed or ambidextrous
• Tested with the Edinburgh Handedness Inventory [13]
12. Dyslexia
13. Claustrophobia
14. Contraindication to MRI scan
15. Non English-language users

^a^Urine sampling for testing for drugs of abuse takes place during the recruitment process. In addition, during study stage 2 (neurobehavioural investigation), urine sampling is repeated prior to starting the trial on day 1 of each of the three study arms, as well as prior to the scanning session on day 5 on each of the three study arms. A seven-drug (multi-panel) dip and read immunoassay is being used, offering high accuracy in detecting cocaine, amphetamine, methamphetamine, tetrahydrocannabinol (cannabis), opiates, barbiturates and benzodiazepines (SureScreen Diagnostics®)

Needle phobia was an additional exclusion criterion during recruitment for stage 1, as 24-hour blood sampling via a cannula is required. For stage 2, volunteers need to be non-dyslexic, right-handed [13] and fluent in the English language, to ensure they can respond to various verbal stimuli included in the behavioural test and/or questionnaires. Participants also need to pass MRI safety screening and must be able to tolerate spending more than one hour inside the MRI scanner bore. Table 1 gives detailed information about the exclusion criteria.

### Recruitment process

For both studies the same recruitment methods have been used. Study adverts briefly describe the scope of the investigation and basic inclusion/exclusion criteria. Posters are being displayed across multiple faculties within the University of Bristol. Electronic adverts are being sent to subjects who have expressed interest in our studies in the past and given permission to be contacted about study opportunities in the future. The study is also publicly advertised on the University of Bristol Department of Psychology webpages, and is included in the monthly newsletter sent to all subscribers of the Psychology mailing list interested in human research (subscription is free to all members of the general public). Advertisement for study stage 2 occurs after completion of stage 1, allowing for optimised treatment schemes (see below) and temporal design of the neurobehavioural investigation.

**Fig. 1:**
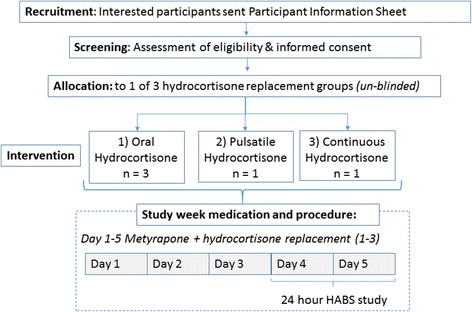
Main steps followed in recruiting and working with volunteers during study stage 1. Interested participants were phone screened, and if screening was positive, an initial appointment was scheduled to discuss in full detail the parameters of the study and answer any queries. During that appointment, a full screening process takes place. If participants are still eligible and willing to participate, signed informed consent is obtained. A date to conduct the five-day interventional study (metyrapone and hydrocortisone administration) is then arranged. The 24-hour study is performed after midday (around 2:00–3:00 pm) of day 4 until after midday (around 2:00–3:00 pm) of day 5. HABS: human automated blood sampler

**Fig. 2:**
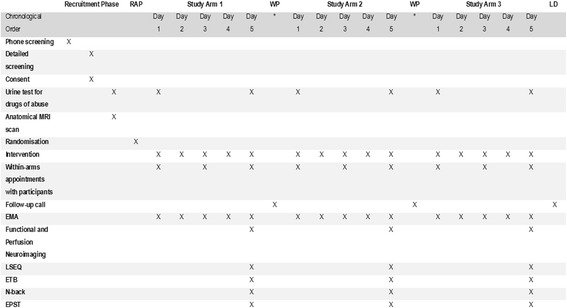
Main steps followed in recruiting and working with volunteers during study stage 2. Interested participants are phone screened, and if screening positive, an initial appointment is scheduled to discuss in full detail the parameters of the study and answer any queries. During that appointment, a detailed screening process would take place. If participants are still eligible and willing to participate, signed informed consent is obtained. Additionally, as part of the screening process, participants need to give a urine sample to test for drugs of abuse and undergo an initial anatomical magnetic resonance (MR) scan. If still eligible, the dates for the conduction of the three study arms are arranged. The order of treatment arm allocation is random and unknown to the volunteers and the study investigators. This is determined by an external authority and takes place prior to the start of arm 1. Interventions during the three study arms were designed and validated during the previous study stage. Each of the three arms takes place with a temporal distance from the previous one of at least two weeks (*). EMA: ecological momentary assessment, ETB: emotional test battery, EPST: emotion potentiated startle test, LD: last study day, LSEQ: Leeds Sleep Evaluation Questionnaire, MRI: magnetic resonance imaging, RAP: randomisation phase, WP: wash-out period

Figures 1 and 2 summarise the study recruitment procedures for both stages of the study. Standard recruitment procedures are used for both stages of the study. Interested volunteers may contact the research team via a secure University email address or telephone number. If the information provided suggests no reason for initial exclusion, subjects are sent the participant information sheet (PIS). The PIS details the aims/scope of the study in lay language, all clinical and experimental procedures involved, the ethical framework which governs the study, the volunteers’ responsibilities and the healthcare and safety information. After receiving the PIS, participants have up to six weeks to consider the information and decide if they would like to take part. If interested, participants are requested to contact the study team by phone or email. Study investigators can also contact potential participants after four weeks if no reply has been received. All interested participants are initially asked to take part in a short screening phone call, arranged at a convenient time. Phone screening allows initial assessment of eligibility and a chance for the investigator to ensure participants have read and understood the PIS and what would be involved in the study. Participants are also encouraged to ask any questions they may have.

If participants are happy to continue and found eligible at this stage, they are invited to attend a more detailed screening visit. During that appointment, a detailed, formal screening process takes place (including the acquisition of a medical history and a physical examination), all the procedures and the scientific background/justification and any medical issues related to the study are discussed and explained in detail. Participants are again encouraged to raise any concerns or queries, and if still willing to take part in the study, they are asked to sign the relevant written consent forms.

Screening for study stage 2 also involves ensuring the participant is suitable for MRI scanning; this is undertaken by using specific screening forms. If it is safe to scan the participant, an additional visit is booked for a preliminary MRI examination during which a high-resolution, anatomical MRI scan is acquired. This initial MRI scan serves two purposes: (1) acquiring the data necessary for subsequent registration of low-resolution functional fMRI data to a “standard” MRI template for neuroanatomical localisation of obtained functional imaging data, and (2) assessing the ability of the subject to lie still inside the MRI scanner bore for long periods. Prior to the scan, participants are also asked to produce a urine sample to test for drugs of abuse (see Table 1).

### Medical issues, quality control, bioethics and trial monitoring

Local ethical and institutional approval has been obtained. The study was discussed with the Medicines and Healthcare products Regulatory Authority (MHRA) and formal approval was deemed unnecessary. The study is conducted according to the principles of the Research Governance Framework for Health and Social Care and the international conference for harmonisation of good clinical practice (ICH GCP, declaration of Helsinki). The study is being performed subject to the Faculty of Science Human Research Ethics Committee of the University of Bristol (REC, ethics approval code: 2706132525) and local Research and Development (R&D) approval of the University Hospitals Bristol (UHB, National Health System Foundation Trust) (reference code: ME/2013/4325). The study is monitored and audited in accordance with the R&D governance procedures of UHB and the University of Bristol. Case record forms are produced, and these and all study-related documents will be made available on request for monitoring and audit by the Trust, University and REC.

Study-related information and informed consent is administered by the investigators. Participants receive information designed to inform them extensively on the topic of the investigation. This includes information regarding the overall study, its goals, methods, health and safety issues and procedures involved. They also receive a detailed presentation of the MRI techniques, their limitations and their safety issues. In addition, during the initial appointment (see above), investigators ensure that participants’ questions and concerns are addressed and fully answered. Participants are informed that they have the right to discontinue their participation at will, at any time without giving a specific reason. Lastly, participants are encouraged to contact the research team at any later point if they feel they require further elaboration on a study-specific topic. Afterwards, eligible male volunteers who are still interested in participating in the study are requested to read and sign a consent form acknowledging the principles outlined above.

The possible presence of unwanted effects due to the pharmacological intervention or other medical processes during the study has been taken into consideration, and all necessary measures, ensuring participants’ safety, have been implemented. For instance, side effects of metyrapone include nausea, vomiting, drowsiness, abdominal pain and allergic skin reactions. For this reason, volunteers are explicitly instructed to accompany pills with food intake to avoid/attenuate gastrointestinal discomfort. If side effects develop, participants are strongly advised to contact the research team, so the metyrapone dose can be decreased for 24 hours and increased again, symptoms allowing.

Potential side effects of (high doses of) hydrocortisone include problems with disturbed sleeping, abdominal pain and heartburn. Since all volunteers will be receiving a cortisol blocking agent (metyrapone) in combination with adequate hydrocortisone replacement therapy, there should be no issues with symptoms related to hypoadrenalism or cortisol excess. For safety reasons, the risks and symptoms of a hypoadrenal crisis are explicitly discussed with participants, as well as what they need to do should they become unwell at all during the study [14]. An emergency box is given to all participants for the duration of their study sessions, containing hydrocortisone for intramuscular and per os administration, as well as other equipment related to the pump handling and subcutaneous delivery. Moreover, all participants have an emergency card to keep on their person at all times with details of study drugs and out-of-hours contact information (24-hour emergency telephone number).

There is a rare possibility of an anaphylactic reaction [15] to parenteral hydrocortisone succinate administration in a subject with no prior history of anaphylaxis. All medical and biomedical procedures related to this study will occur in the Joint Clinical Research unit at UHB or the Clinical Research and Imaging Centre of the University of Bristol (CRICBristol). Both units’ basic resuscitation equipment has been inspected and agreed to be appropriate by the UHB resuscitation officer, and there is immediate access to the full resuscitation team if they are needed. A standard operating procedure and risk assessment for the procedure includes clear instructions regarding the presentation and initial management of anaphylaxis.

In case of technical, pump-related and drug delivery-related issues, such as subcutaneous pump line falling out/accidental removal, all participants are taught how to perform a line change (materials provided in the emergency box) and instructed to contact a study researcher (24-hour emergency telephone number provided) so that the pump can be checked and a replacement emergency kit provided. In case of a pump failure/malfunction, subjects are instructed to contact a study researcher. If a replacement pump cannot be provided within 3 hours, the subject will be asked to take oral hydrocortisone replacement (provided in the emergency box).

Other minor unwanted effects that may occur during the study are discomfort, bruising, infection or allergic reaction at site of insertion of infusion pump/cannula, vasovagal reaction or bruising due to intravenous cannulation (during the preliminary stage of the study), and a feeling of claustrophobia during MRI scanning (during the core part of the study). Adverse events will be recorded in accordance with the ICH GCP and the UHB’s research-related adverse event reporting policy. The study will be stopped prematurely in the case of significant adverse events.

Concerning the MRI scans, the subjects are made aware that these scans are not diagnostic and will be interpreted solely for the purposes of the study. Nevertheless, study researchers do have a duty of care. As a consensus between these two principles, this study has adopted the guidelines provided by Illes et al. [16], which explain the procedures for dealing with incidental findings in brain imaging research. Based on these guidelines, our scanning protocol (see below) contains the minimum required sequences for research and does not include extra scans for diagnostic purposes. All scans are reviewed by a clinically qualified (non-radiologist) member of the research team. If any concerns are noted, the images will be sent for consultation and/or referral to a radiologist for clinical evaluation. After assessment the qualified radiologist will report any incidental findings to the lead researcher and provide guidance on what should be conveyed to the participant’s primary care provider (this information is collected at screening). After reporting, the responsibility for assessing the importance of the findings and acting on them rests with the primary care provider.

Volunteers are free to withdraw from the study at any time. Should a subject withdraw from the study, another will be recruited in his/her place. It is not anticipated that withdrawn volunteers will require any follow-up.

With regards to a randomisation and/or blinding code break during the second stage of the study, a copy of the codes will be kept in the Bristol Royal Infirmary University Hospital Pharmacy and in the site file. The codes may only be broken under the following conditions with the agreement of the chief investigator and REC except under the case of an emergency: (i) it is deemed clinically necessary (for example, treatment of an adverse event is dependent upon knowledge of the drug administered), (ii) the study must be terminated for safety reasons, or (iii) a third party requires the information (for example, a coroner).

### Study stage 1: optimising and validating pharmacological interventions

The aim of the preliminary stage of the study is to define and validate (technically and pharmacologically) safe methods to create reliably and reproducibly three different patterns of plasma cortisol. These patterns were based on the combinatory administration of metyrapone and hydrocortisone. Metyrapone was chosen to suppress endogenous cortisol production for both safety reasons (as it has a short half-life [17]) and because it does not interfere with cortisol’s molecular physiology. Metyrapone blocks cortisol synthesis by inhibiting steroid 11β-hydroxylase [18]. These three methods for creating different patterns of systemic cortisol dynamics in healthy individuals offered the basis for the temporal design of study stage 2 (see below).

Five healthy volunteers participate in stage 1. After giving written consent, they undergo a five-day treatment scheme, where metyrapone per os (on a full stomach to minimise gastric side effects) treatment is gradually increased from 0.5 g on day 1 to 2.5 g on day 4 (see Table 2). Hydrocortisone replacement is achieved in one of three modes: (i) either orally to mimic the current therapeutic protocols for glucocorticoid replacement or via a subcutaneous pump, delivered (ii) continuously or (iii) in pulses. The total daily dose of hydrocortisone is: 20 mg (per os), 19.8 mg (continuous with varying rhythm throughout the day) [19], 19.9 mg (pulsatile: eight times/day being a close approximation of the adrenal glucocorticoid natural ultradian pattern of secretion) (see Table 3). For technical reasons, hydrocortisone delivery via the pumps differs very slightly from the oral 20 mg dose. We have chosen to deliver slightly less by subcutaneous infusion to compensate for the slightly higher blood levels achieved using this route [20, 21]. Recently, our group has published the technical data (dose range and frequency of individual pulses estimation) and the functional validation of the pulsatile pump [22].

**Table 2:**
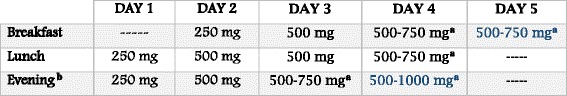
Outline of the five-day metyrapone treatment scheme during study stage 1

Medication was received orally (pills of 250 mg of metyrapone). The doses in blue letters have been administered during the 24-hour blood sampling study

^a^For the purposes of the optimisation procedure, various doses were tested in different subjects at particular time points during the five-day treatment scheme to secure effective adrenal gland suppression (see Fig. 4)

^b^The last dose of the day was shifted from, initially, dinner time to just before going to sleep to ensure effective adrenal gland suppression during sleep (see Fig. 4). Participants are strongly advised to take metyrapone with a glass of milk or a snack to avoid any gastric discomfort

**Table 3:** Outline of the three different modes of hydrocortisone replacement in healthy individuals under metyrapone administration

Time of day	Per os	SC continuous	SC pulsatile
00:00		0.1 mg/hour	0.5 mg
01:00			
02:00		2 mg/hour	
03:00			4 mg
04:00			
05:00			
06:00			4 mg
07:00	After waking up (10 mg)		
08:00		1 mg/hour	
09:00			4 mg
10:00			
11:00			
12:00		0.4 mg/hour	2.3 mg
13:00	During lunch (5 mg)		
14:00			
15:00			2.3 mg
16:00			
17:00			
18:00			2.3 mg
19:00	During dinner (5 mg)		
20:00		0.1 mg/hour	
21:00			0.5 mg
22:00			
23:00			

The last two modes involve the subcutaneous (SC) delivery of hydrocortisone via a pump; the continuous pump (Animas® Vibe™ Insulin Pump) is programmed to constantly deliver hydrocortisone but with a changing flow rate throughout the day. The pulsatile pump (Crono P®, CANE Applied Medical Technology Ltd, Cambridge, UK) is programmed to deliver a certain amount of hydrocortisone every three hours with a rhythm of 10 μL/s [22]

All visits take place in the Joint Clinical Research unit (UHB). On day 1 participants are fitted with the subcutaneous pump and are given precise instruction on how to receive their oral medication. On day 3 participants wearing either of the pumps attend the unit for a pump syringe and line change. On day 5, the 24-hour blood sampling study is undertaken, using the human automated blood sampler (HABS). Blood samples are collected via an intravenous cannula every one hour for serum corticotrophin (ACTH) and cortisol concentration estimation. For those receiving hydrocortisone via the subcutaneous pulsatile pump, cortisol was being estimated every 10 minutes, since we anticipated that cortisol would fluctuate in a more dynamic manner (approximating its normal, ultradian rhythm). During the 24-hour sampling procedure, participants give approximately half a pint of blood (half the amount of blood taken at blood donation). All blood is replaced with an equal volume of normal saline.

During the blood sampling period, participants are served a hot meal at 7:00 pm, breakfast at 07:00 am and lunch at 12:30 pm. Refreshments are offered between meal times and recorded. Participants are asked to go to sleep at 11:00 pm when the lights are switched off. During the lights-on period, participants are allowed to work, read, watch films or relax. A member of staff is present in a room adjacent to the experimental room to monitor them at all times. At the end of the study (in the early afternoon), when the intervention is terminated, participants are instructed to take orally 5 mg of hydrocortisone during dinner to ensure proper cortisol replacement for that day. This ensures there has been adequate time for the metyrapone to have cleared and resumption of endogenous cortisol production. Participants are called the following day to check that there have been no adverse events and note any issues they wish to report.

Cortisol samples were allowed to clot at room temperature prior to centrifugation and serum was frozen at −80 °C until assayed. Samples for ACTH were collected in chilled ethylenediaminetetraacetic acid-containing tubes and kept on ice until centrifugation at 4 °C within 30 minutes. Plasma was stored at −80 °C until assayed. Analysis is performed by the department of clinical biochemistry at the UHB using an electrochemiluminescence immunoassay (Cobas®, Roche). Cross-reactivity with 11-deoxycortisol was 4.1 %. This immunoassay has an intra-assay coefficient of variation ranging from 1.5 % to 6.1 % on sample concentrations of 0.170 μg/dL to 0.718 μg/dL http://www.accessdata.fda.gov/cdrh_docs/reviews/K043175.pdf.

### Study stage 2: randomisation and blinding

The second stage of the study is the randomised, double-blinded, placebo-controlled, three-way crossover investigation, where participants undergo all three optimised five-day treatment schemes as finalised in the previous stage. Randomisation schedules were generated using IBM SPSS® v19 software by staff members not directly involved in data collection for this study. Dispensing and processing of all medication/placebo is managed by Bristol Royal Infirmary University Hospital Pharmacy, ensuring that study researchers remain blinded to the conditions. To ensure that the study remains double-blind, all participants are required to take the same daily regime of tablets and remain connected to a subcutaneous pump (continuous or pulsatile). Each week one of these treatments will be placebo (either placebo tablets or 0.9 % saline infusion via the pump). In addition, the study investigators responsible for care of the pumps have no input into the psychological testing. There is a minimum of two weeks between each of the three study arms. Participants and study researchers are therefore unaware of the order of the arms of their hydrocortisone replacement.

On day 1 of each arm, participants are connected to the pump via a line that is inserted into the subcutaneous tissue of the abdomen and are trained to handle the pump by themselves (detach-reattach). Participants are given metyrapone and identically encapsulated tablets containing either hydrocortisone or placebo and the investigator explains to them the five-day treatment plan they will follow. The participants receive instructions in cases of an emergency (hypoadrenal symptoms). Finally, they also receive their study android phone that monitors their everyday state of mood and reactivity at random points within each day (see below). Participants also give a urine sample to be tested for the presence of drugs of abuse.

On day 3 participants attend the hospital for a pump syringe and pump line change, while the investigator also monitors individuals’ compliance to the treatment scheme (counting the number of remaining pills) and to answering the study phone’s questions. Finally, on day 5 participants attend CRICBristol where they undergo MRI scanning, complete a sleep questionnaire and undertake a series of behavioural tests (see below). At the end of the study visit, participants return all study material (android phone, remaining pills), are disconnected from the pump and are instructed to take 5 mg of hydrocortisone orally during lunch and again during dinner to ensure proper cortisol replacement for the remaining day. This ensures there has been adequate time for the metyrapone to have cleared and resumption of endogenous cortisol production. They will then receive a follow-up phone call the following day to check to see how they are feeling and if they have any issues they wish to report.

Compliance to the treatment protocol is encouraged by (1) giving participants at arm day 1 a detailed, written five-day timetable, with instructions on the timings and the number of metyrapone and hydrocortisone/placebo pills they need to take, (2) counting the remaining pills at arm days 3 and 5 to verify that they are consumed in the proper quantity, and (3) prompting the participants at arm day 3 to accurately follow their treatment scheme and to constantly wear the pump. Furthermore, compliance to the oral treatment protocol is also supported via three reminder messages (in the morning, at 1:00 pm and at 7:00 pm) delivered every day through the study phones carried by each subject.

### Assessments during study stage 2

#### Ecological momentary assessment

This is a subjective test that relies on the repeated, five-day long collection of real-time data on subjects’ behaviour/emotional status in the subjects’ natural environment. For this purpose, volunteers are asked to continuously carry a modified android mobile phone, for the whole duration of each of the three five-day study periods. This is appropriately programmed to ask some questions contained in the Identity-Consequence Fatigue Scale [23] and visual analogue scale [24] concerning the subjects’ state of reactivity and feeling of well-being. Time-based sampling is applied; assessments are solicited based on a time schedule, but at random time intervals. Additionally the phone reminds the holder to take his oral study medication. Moreover, the device asks the subject to complete a morning (available until around 10:00 am) and an evening report (available at 07:00 pm onwards) each study day. Anonymised data (subjects’ feedback), both quantitative and qualitative, are stored in the android phone, and are transferred to University of Bristol computers.

Ecological momentary assessment (EMA) techniques (versus static retrospective self-reports of behaviour) take into consideration the dynamic changes in behaviour over time and across situations and under a real-world setting. Moreover, they avoid relying on individuals’ autobiographical memory (which is the case in retrospective self-reports), which is prone to random error but also fraught with systematic bias [25]. Evidence from other clinical studies, such as smoking cessation, indicates that ambulatory monitoring of subjective states provides greater temporal resolution than can be achieved by laboratory assessments [26, 27]. This setting provides rich and detailed information on both the background level of mood and well-being (tonic levels), as well as the frequency and magnitude of acute fluctuations (phasic variation), and identifies specific periods when these occur (for example, early morning). EMA data offer the opportunity to evaluate hypotheses regarding the dynamic interactions among processes over time—in our case, how different systemic cortisol dynamics could affect reactivity, mood and well-being perception [28, 29].

#### Leeds Sleep Evaluation Questionnaire

During day 5 of each study arm participants are asked to complete the Leeds Sleep Evaluation Questionnaire (LSEQ). This scale comprises ten self-rating 100-mm-line visual analogue questions evaluating four main components: sleep initiation, sleep quality, ease of waking and behaviour following waking. Anonymised data is transferred to the University of Bristol computers. Application of the LSEQ offers consistent and meaningful findings concerning subjectively perceived changes in sleep during psychopharmacological investigations, offering good retest reliability, cross-cultural validity and stability across a range of clinical settings [30–32]. Moreover, it has recently been used to correlate basal diurnal salivary cortisol profiles with sleep quality [33].

#### P1vital Oxford behavioural assessments

Participants undertake a series of emotion-related experimental paradigms, primarily designed to measure biases in emotional processing that are thought to be core components of depression [34]. The P1vital® Oxford Emotional Test Battery (ETB) consists (in our study design) of four paradigms consecutively run during one session which takes place during the fifth day per study arm. The paradigms follow the same order as presented below. The Facial Expression Recognition Task (FERT) assesses the interpretation of facial expressions. Faces of six different basic emotions (happiness, fear, anger, disgust, sadness, surprise) are displayed on the screen and subjects are required to indicate the expression of the face via a button-press. Different intensity levels of each emotion are presented, which increases the ambiguity of the facial expression and the sensitivity of the task. The Emotional Categorisation Task (ECAT) assesses speed to respond to positive and negative self-referent personality descriptors. Sixty personality characteristics selected to be disagreeable or agreeable are presented. The subjects are asked whether they would like or dislike to be referred to as each characteristic. The Faces Dot Probe Task (FDOT) assesses attention to positive versus negative stimuli using a reaction time measure. Two faces are presented vertically on the computer screen and replaced by a pair of dots, to which the subject has to respond by indicating whether the dots are vertically or horizontally aligned. During some trials, one of the two faces presented has an emotional expression (fearful or happy). The reaction time to respond to the dots presented in the same or different location to the face stimuli can be used as a measure of attention to the emotional faces. The stimuli are presented either masked (briefly and replaced by a scrambled face stimulus) or unmasked (subject to conscious awareness). Lastly, the Emotional Recall Task (EREC) is a surprise free recall task to assess the incidental encoding of emotional stimuli. Subjects are asked to recall as many of the words previously presented in the ECAT task as they can. The relative recall of positive versus negative words gives a measure of emotional biases in memory. Contrary to all previous tasks, the latter one is not computerised (subjects write the recalled words on a piece of paper).

Another behavioural test that participants undergo during the fifth day of each study arm is the P1vital® Oxford Emotion Potentiated Startle Test (EPST). EPST is designed to provide an objective measure of response to different emotional stimuli [35]. Subjects are exposed to three consecutive blocks of visual stimuli (= photographs from the International Affective Picture System database with positive, negative or neutral content), while being synchronously exposed to temporally random acoustic stimuli (startle-eliciting pulses through white noise). Startle responses are recorded using electromyography of the orbicularis oculi muscle. Electromyographic data are stored anonymously in the University of Bristol electronic databases.

FERT, ECAT, FDOT, EREC and EPST have been used in previous behavioural and fMRI studies and have been established as models of emotional and cognitive processing sensitive to the effects of neuropsychopharmacological interventions [36]. Moreover, the test-retest reliability of these tasks is good for the purposes of this crossover study, with the exception of FERT where performance of healthy subjects was found improved one week after a baseline test, though the relative effect of specific emotions was unaltered [37]. To overcome this issue and despite the fact that our test-retest period will be longer than a week, we have introduced different facial stimuli per testing session.

#### N-back

During the fifth day of each study arm, participants are asked to undertake a cognitive test measuring aspects of working memory. In this task, a series of letters will appear on the screen one at a time. The participant needs to make a response as to whether the letter that he sees each time on the screen matches a letter presented previously (= reference letter). This continuously changing reference letter depends on instructions that appear at the beginning of each of the many letter sequences. If the appearing letter matches the reference letter, he should press a button labelled as “same”, and in all other cases a button labelled as “different”. In the one-back task, the reference letter is every previous letter, in the two-back test the reference letter is every second previous letter and in the three-back test every third previous letter. All data are anonymously stored in the University of Bristol computers.

The n-back task captures the active part of working memory. When n equals 2 or more, it is not enough to simply keep a representation of recently presented items in mind; the working memory buffer needs also to be updated continuously to keep track of what the current stimulus must be compared to. To accomplish this task, the subject needs to both maintain and manipulate information in working memory. This task has been shown to activate frontal and parietal cortical regions, among them the dorsolateral prefrontal cortex [38]. Moreover, the n-back has a good test-retest reliability in terms of the neural processing recruited for this cognitive task [39], while behaviourally, daily practice for a duration of weeks is required to gain any significant benefits in performance [40] independent of any experimental conditions.

#### Functional and perfusion imaging

**Fig. 3:**
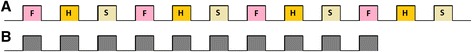
Tasks of controlled block design used during the functional neuroimaging protocol. **a** During the implicit facial expression processing task, individuals are exposed to alternating visual stimuli (pictures) consisting of 30-second blocks of human faces with a particular facial expression (fear -F- or happiness -H- or sadness -S). Each block corresponds to one kind of facial expression, where 10 different human faces, male or female, with the same facial expression are presented for 0.1 second with a 2.9-second inter-stimulus interval. Blocks are divided from each other by resting state periods (lasting 30 seconds). Each kind of block (F, H, S) is repeated four times. The paradigm starts and finishes with a resting state period. Participants are given a button box and explicitly instructed to press a corresponding button depending on whether the face they see each time is male or female (gender discrimination). Both accuracy and response time are recorded. **b** During the flashing checkerboard task, individuals are visually exposed to a flashing checkerboard (a checkerboard whose cells alternate between white and black colours with a frequency of 7.5 Hz) for 16 seconds followed by a resting period of 15 seconds before the next flashing checkerboard visual stimulation initiates. The resting period-flashing checkerboard alternation is repeated 10 times. Subjects are instructed to have their eyes open and look at the screen all the time

During the fifth day of each study arm, participants undergo a specific brain MR scanning protocol. The protocol consists of two block-designed task-based fMRI paradigms (see Fig. 3), a resting state fMRI, a pseudo-continuous arterial spin labelling-based perfusion imaging scan (using six post-labelling delays; 0.25 s, 0.50 s, 0.75 s, 1.00 s, 1.25 s, 1.5 s), as well as a number of supportive MRI sequences. The temporal design of the MR scanning protocol is based on the results of study stage 1 (see below and Table 4). Table 5 presents some technical details about the MRI experiment, following the guidelines for reporting an fMRI study, as presented in Poldrack et al. [41]. To confront with the low test-retest reliability of the facial expression processing task [39], as with FERT, we are introducing different facial stimuli per testing session. Note that the mean test-retest period is anticipated to be larger than 14.6 days, which is the one tested in Plichta et al. [39].

**Table 4:** Temporal design of study stage 2 and the concurrent relative glucocorticoid dynamics per study group

		Cortisol dynamics		
		Ultr.	Circ.	PO
MRI scanning protocol	08:45–10:00 am			
• Localizer	08:45–09:05 am	*D*	*S*	*D*
• TOF_3D_Neck	08:45–09:05 am	*D*	*S*	*D*
• EPI_2D_Resting State	08:50–09:10 am	*S*	*S*	*D*
• EPI_2D_Zshim_calibration	09:00–09:15 am	*A*	*S*	*D*
• EPI_2D_Zshim_IFEPT	09:05–09:30 am	*A*	*S*	*D-S*
• EPI_2D_pCASL	09:20–09:45 am	*A*	*S*	*D-S*
• GRE_field mapping	09:35–09:50 am	*A*	*S*	*D-S*
• EPI_2D_Zshim_FCT	09:40–09:55 am	*S*	*S*	*D-S*
LSEQ	10:00–10:20 am	*D*	*S*	*S*
Oxford P1vital ETB	10:15–10:50 am	*D*	*S*	*S*
N-back	11:10–11:40 am	*D-S*	*S*	*S*
Oxford P1vital EPST	12:05–12:50 pm	*A*	*S-D*	*S-D*

Non-task scans will include perfusion imaging (pCASL), supportive imaging to reduce geometric distortions (Z-shimming calibration and field mapping) to allow for increased accuracy of analysis, and resting state imaging to assess functional connectivity. The EPI_2D_Zshim_calibration sequence is specifically designed to reduced signal dropout from the orbitofrontal cortices. 2/3D: two-/three-dimensional, *A* ascending cortisol levels, *pCASL* pseudo-continuous arterial spin labelling, *Circ.* circadian (without the ultradian component), *D* descending cortisol levels, *EPI* echo planar imaging, *ETB* emotional test battery, *EPST* emotion potentiated startle task, *FCT* flashing checkerboard task, *GRE* gradient recalled echo, *IFEPT* implicit face expression processing task, *LSEQ* Leeds Sleep Evaluation Questionnaire, *PO* per os, *S* steady (no significant changes in) cortisol levels, *TOF* time-of-flight, *Ultr.* ultradian and circadian rhythm, *Zshim* shimming along the Z axis

**Table 5:** Technical image properties of the three functional MR sequences used for data acquisition

	MRI system		
	Siemens Magnetom Skyra syngo MR D13C (3 tesla)		
	MRI acquisition details		
	IFEPT	FCT	RS
Volumes	250	105	180
Pulse sequence type	EPI	EPI	EPI
FOV	192 mm	192 mm	192 mm
Matrix size	64x64 mm	64x64 mm	64x64 mm
Slice thickness	3 mm	3 mm	3.5 mm
Acquisition orientation	T > C-30.0 > S2.3	T > C-30.0 > S2.3	T > C-30.0 > S2.3
Whole brain?	Yes	Yes	Yes
Bandwidth	2298 Hz/Px	2298 Hz/Px	2170 Hz/Px
Order of acquisition	Interleaved	Interleaved	Interleaved
TE	30 ms	30 ms	28 ms
TR	3,000 ms	3,000 ms	2,040 ms
Flip angle	87°	87°	89°

*C* coronal, *EPI* echo planar imaging, *IFEPT* implicit face expression processing task, *FCT* flashing checkerboard task, *FOV* field of view, *RS* resting state imaging, *S* sagittal, *T* transverse (axial), *TE* echo time, *TR* repetition time

The determination of the brain regions of interest (ROIs) in our study is based on the following criteria: (1) areas which are glucocorticoid-sensitive (that is, express glucocorticoid and mineralocorticoid receptors) according to the most up-to-date knowledge [42–46], while at the same time (2) are implicated in (face) emotional processing and mood regulation [47–51]. Based on the above criteria, eight ROIs have been pre-selected: amygdala, prefrontal cortex (with particular focus on orbitofrontal cortex), insular cortex, dorsal striatum (part of basal ganglia), nucleus accumbens (part of ventral striatum), hippocampal formation, and anterior and posterior cingulate cortex.

### Power analysis: how many subjects in study stage 2?

With a sample size of 15 participants on whom complete data are available, we will have 80 % power at an alpha level of 5 % to detect an effect size equivalent to f = 0.35. This is our current best estimate of the likely effect size across the three conditions of our study, based on work in other settings using pharmacological challenge (for example, antidepressants) to probe these psychological mechanisms.

### Data analysis for study stage 2

For the EMA data, the area under the curve will be calculated for measures of tiredness and mood. These will be used as the primary outcome variable. Secondary analyses will be conducted on other derived variables, such as the degree of variability in tiredness and mood over time (that is, amplitude). In ETB and n-back tasks, interpretation of the neurobehavioural phenotypes will result from co-evaluating the percentage of correct and false responses, the mean latency of correct responses (in milliseconds) and percentage of non-responses among the different study groups [35, 52]. The recorded task measures of accuracy, reaction times, and — where relevant — signal magnitude will be grouped according to relevant stimulus properties such as valence (positive/neutral/negative) and analysed for effects of pharmacological treatment using analyses of variance (ANOVA). In EPST, the presence and magnitude of eye blinking in response to white noise will also be grouped according to relevant stimulus valence (positive/neutral/negative) and compared across the three different groups using ANOVA. This will also be done in LSEQ.

At the individual level, functional imaging data will be pre-processed to remove the influence of external factors like magnetic field inhomogeneities and motion artefacts, extract the brain from the skull and other soft tissues, co-register different imaging modalities (low-resolution functional images with the corresponding high-resolution anatomical image) with a standard reference template (MNI152). Prior to model estimation, the time series blood oxygen level-dependent (BOLD) data will be temporally filtered (high pass) and spatially smoothed in an effort to improve the signal-to-noise ratio. A general linear model will be applied to the task-based fMRI data to estimate BOLD signal change associated with each paradigm. Subsequent group effects will be assessed via mixed effects inference with a Bayesian two-level model with fast approximation to posterior probability of activation using Functional Magnetic Resonance Imaging of the Brain (FMRIB) Software Library (FSL) software [53] in combination with statistical parametric mapping (SPM) software [54]. Other techniques will be recruited to explore functional connectivity of the brain.

## Discussion

This project aims to investigate the impact of dynamic changes in glucocorticoid rhythmicity on neural processing of emotional perception as well as cognitive and behavioural phenotypes in healthy male individuals, utilising functional imaging of the brain and a series of cognitive and psychological tests. To test this we are using a double-blind, three-way crossover randomised design. This will allow us to perform within-subject analysis, comparing three methods of hydrocortisone delivery: oral, (subcutaneous) continuous and (subcutaneous) pulsatile.

Determination of the inclusion and exclusion criteria was based on a number of known neuroendocrinological and neurophysiological factors that could alter HPA axis function and bias results. Nicotine, alcohol and caffeine [55–57] are known to elevate cortisol and are carefully controlled for at screening and then during the study. Concurrent use of other prescribed drugs (especially steroid-based) or recreational substances may also influence HPA axis function [58], and may confound functional neuroimaging data [59].

Only male individuals were recruited for this study, as there is a sexually dimorphic difference in the behaviour in response to cortisol [60, 61] and functional variations in the neuroanatomical distribution of glucocorticoid-mediated cognitive and emotional processing [62, 63]. In relation to our study, a recent meta-analysis [64] has indicated a sex-dependent difference in the effect size of the BOLD signal response of amygdala after exposure to emotional faces, which is one of the main fMRI paradigms we use. In addition, response of female individuals to hydrocortisone is modulated by oral contraceptive use and varies over the menstrual cycle [65], with the latter being difficult to control for, given the crossover nature of the study.

In this study the control of handedness is also important, as there is evidence for a positive relationship between degree of handedness and degree of cerebral lateralisation for processing emotional faces in men [66]. Dyslexia has been related to impaired processing of rapid stimulus sequences [67] similar to the ones used in our study, and was therefore controlled for.

The issue of cultural differences and the extent of universality in emotional perception is very complex. In general, happiness, anger and (dynamic) non-verbal channels of communication are parameters that show less cross-cultural variability (that is, are characterised by a lesser within-cultural-group advantage) [68]. During the design of study stage 2, we avoided setting cultural/racial criteria in our recruitment strategy for ethical reasons; rather we chose to primarily rely on investigational tools of emotional perception that show the least possible cross-cultural variability.

General intelligence was not controlled for in this study, as there is no convincing evidence suggesting that the degree of general intelligence is linked to particular patterns of brain morphology [69] or frontal and corticolimbic activation patterns under emotional stimulation [70], which are our regions of interest. Other individual differences (intrinsic environmental and genetic factors) could not be assessed and consequently could not be controlled.

All three methods for creating different daily patterns of circulating glucocorticoids are based on the combined treatment of metyrapone and hydrocortisone. This offers a direct relevance for the treatment of actual patients with hypoadrenalism. Thus, this study could potentially also provide pilot data on whether replication of the ultradian pattern in glucocorticoid-based therapeutics could improve treatment efficiency and attenuate side effects. Even in the case of adrenal insufficiency, replacement therapy under the established therapeutic protocols is accompanied by a higher mortality rate, increased morbidity from poor quality of life (mental and physical fatigue, cardiovascular, malignant and infectious diseases), increased psychosocial needs and morning fatigue compared to normal individuals [71]. The presence of neuropsychiatric symptomatology, and all other iatrogenic problems, in these patients suggests that temporal aspects of glucocorticoid therapy may be helpful in achieving their homeostatic effects rather than their deleterious side effects.

The optimisation process for our metyrapone blockage and hydrocortisone replacement therapy is based on two criteria: (1) the preservation of systemic cortisol levels within a physiological range during hydrocortisone replacement, and (2) the suppression of the endogenous adrenal activity during the 24-hour studies. Endogenous adrenal activity can be biochemically discriminated from exogenous hydrocortisone administration in these participants due to the prolonged interval (10–12 hours) between the last per os hydrocortisone dose of day 4 (around 07:00 pm) and the first hydrocortisone dose of day 5 (around 07:00 am). Based on the above criteria, we have derived the ideal metyrapone treatment dose for study days 4 and 5 as: 0.75 g during breakfast (day 4), 0.75 g during lunch (day 4) and 1 g before going to sleep at night (day 4) and 0.75 g during breakfast (day 5). This treatment strategy effectively suppresses endogenous adrenal activity in a reversible manner and results in plasma cortisol profiles that reliably reflect the pattern of exogenous hydrocortisone administration. This is also confirmed with the subcutaneous hydrocortisone replacement [19, 22] strategies (see Figs. 5 and 6) in two other healthy male volunteers. None of these five participants reported any side effects (expected or unexpected).

**Fig. 4:**
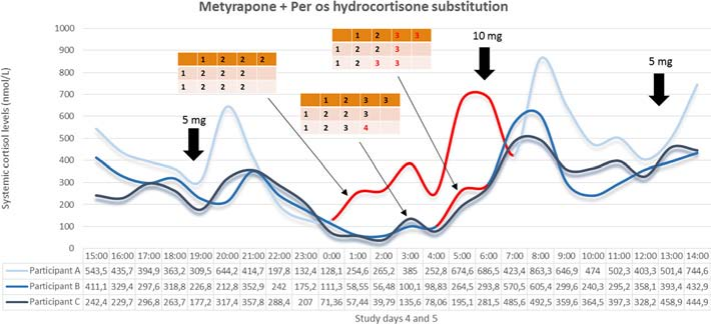
Systemic 24-hour cortisol profiles of three individuals participating in study stage 1. All participants were under the combined treatment of metyrapone and hydrocortisone per os. The pattern of oral hydrocortisone administration is presented in Table 3, and was identical in all three participants. Metyrapone treatment differed between the three individuals as indicated in the corresponding small tables within the figure; for each small table, each column represents a study day and each row represents the time point within the day in chronological order (during breakfast, lunch and in the evening) to receive metyrapone. The numbers within the small tables’ cells represent the number of metyrapone pills administered (1 pill = 250 mg metyrapone). Circulating cortisol levels in participant A exceeded normal values at all periods of his 24-hour study, while during his sleep period cortisol pulses were present (red part of the curve) which could not be explained by substitution therapy. Thus, adrenal gland suppression was ineffective. For participant B, the evening dose at every study day was transferred from dinner time to just prior to going to sleep for the night, and a number of doses during study days 3, 4 and 5 were increased from 2 to 3 pills, as indicated in red colour in corresponding small table. Circulating cortisol levels in participant B remained under normal values at all periods of his 24-hour study except for the morning peak and some signs of endogenous adrenal activity just prior to awaking (red part of the curve). For participant C, the evening dose of study day 4 was further increased from 3 to 4 pills as indicated in red colour in corresponding small table. Based on the biochemical results of participant C, this last metyrapone treatment scheme was adapted as the ideal one to effectively suppress adrenal gland for the exogenous hydrocortisone administration to reliably define the exogenously derived pattern of circulating cortisol dynamics

**Fig. 5:**
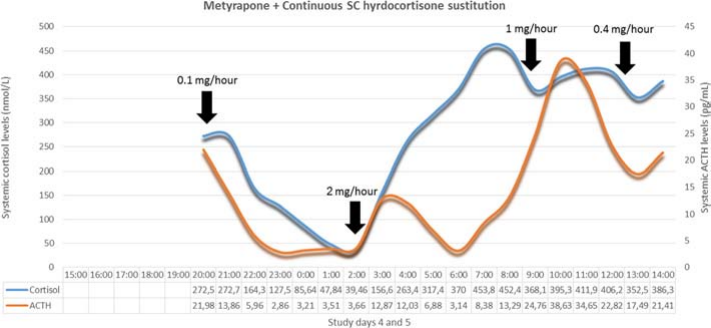
Optimised metyrapone treatment and subcutaneously continuous hydrocortisone replacement. Plasma 24-hour cortisol and corticotrophin (ACTH) profiles of one individual participating in study stage 1. The subject received the optimal metyrapone (Fig. 4, participant C) along with hydrocortisone substitution therapy, subcutaneously (SC), in a continuous manner via Animas® Vibe™ Insulin Pump. The pattern of continuous SC hydrocortisone administration is presented in Table 3; between 08:00 pm–02:00 am the flow rate of hydrocortisone substitution is 0.1 mg/h, followed by an increase to 2 mg/h between 02:00–08:00 am, to drop to 1 mg/h between 08:00 am–12:00 pm, followed by a further decline to 0.4 mg/h between 12:00–08:00 pm. (Total daily dose adds up to 19.8 mg/day.) The black arrows indicate the time points of shifting from one flow rate to the next. Due to technical problems, blood samples of the first 5 hours of the study could not be analysed. This mode of hydrocortisone replacement tries to mimic the normal circadian profile (daily cortisol levels rise in the early morning hours reaching their peak around 08:00 am, near 500 nmol/L before starting to fall throughout the rest of the day to reach their trough around 02:00 am of the next day, near 50 nmol/L) but without the physiologically underlying ultradian rhythm. ACTH fluctuations within normal values confirm the physiological state of the hypothalamic-pituitary-adrenal axis.

**Fig. 6:**
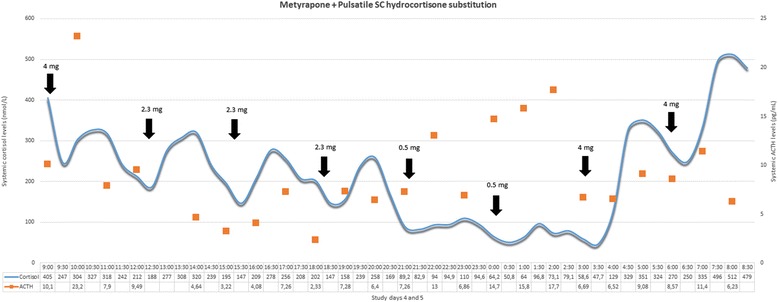
Optimised metyrapone treatment and subcutaneously pulsatile hydrocortisone replacement. Plasma 24-hour cortisol and corticotrophin (ACTH) profiles of one individual participating in study stage 1. The subject received the optimal metyrapone (Fig. 4, participant C) along with hydrocortisone substitution therapy, subcutaneously (SC), in a pulsatile manner via Crono P® pump (CANE Applied Medical Technology Ltd, Cambridge, UK). The pattern of pulsatile SC hydrocortisone administration is presented in Table 3; pulses are also indicated as black arrows in the figure. Corresponding dose of hydrocortisone is indicated above each arrow (adding up to 19.9 mg/day). This mode of hydrocortisone replacement tries to approximate the physiological (normal circadian and underlying ultradian) profile of endogenous cortisol secretion. ACTH fluctuations within normal values confirm the physiological state of the hypothalamic-pituitary-adrenal axis

During the neurobehavioural investigation, 15 participants will undergo all three methods of hydrocortisone delivery for five days as optimised during the previous study stage. EMA via the android phone will gather daily data about self-perceived mood and emotional reactivity of each subject, during these five-day periods. On the final day of each study arm (day 5), participants will undergo a number of neurobehavioural tests (see Table 4). Based on the biochemical results of the preliminary stage of the study (Figs. 4, 5 and 6), the MR scanning will take place between 8:45–10:00 am. During this period of time glucocorticoid dynamics among the three study groups show distinctly different motifs (see Table 4). The EPST will take place after 12:00 pm to capture the effect of the pulsatile mode of replacement which results in a rising phase in the subjects’ plasma cortisol levels in contrast to the other two study groups. Overall, as seen in Table 4, the exact time periods during which participants are scanned and tested during arm day 5 of study stage 2 correspond to specific phases of circulating cortisol dynamics per treatment strategy.

A last point worth mentioning concerns the study groups selected for this study. Theoretically, two extra groups could have been added to the investigational framework: one group of minimal glucocorticoid effect (for instance, metyrapone-only treatment) and a “control group” with no treatment intervention. Both of these groups would have been problematic from a scientific point of view. A metyrapone-only treated group would render otherwise healthy (but for the purposes of the study hypoadrenal) subjects prone to life-threatening hypoadrenal crisis. In addition, metyrapone would not only deplete the presence of glucocorticoids in the brain but would also enhance precursors such as 11-deoxycortisol and 11-deoxycorticosterone, which have been recently shown to provoke neuromodulatory effects [72]. On the other hand, little would be known about the temporal pattern of circulating cortisol in the control group, as this would be different in each participant. Thus, the psychological tests and fMRI scanning would be performed at random times during the subjects’ ultradian cycles, preventing the ability to place them into a biological context and interpret them in relation to physiology.

Any data extracted from this study will be subject to some limitations. Compliance of participants to the various treatment strategies, though monitored in multiple ways, will depend on their personality and motivation. Moreover, glucocorticoid effects on the brain present gender- and age-related differences [11]; thus, the results will not be necessarily generalizable. Due to technical reasons, we are not able to monitor the dynamic cortisol fluctuations during the neurobehavioural outcome measures as is achieved during the preliminary stage. Thus, during study stage 2, cortisol dynamics will be based on the 24-hour profiles determined during study stage 1. Concerning functional neuroimaging data interpretation, the limitations in spatially correlating activation areas with underlying neuroanatomy, as well as the synaptic rather than neuronal activity-related origin of the BOLD signal [73], must be taken into consideration.

In summary, this study aims to shed light on the impact of different patterns of daily glucocorticoid dynamics on neural processing, emotional reactivity and perception, mood and self-perceived well-being. For this purpose, the study adopts an interventional approach based on per os metyrapone treatment, suppressing in a safe manner endogenous cortisol production, coupled with three alternative patterns of exogenous hydrocortisone replacement. Outcome measures include a range of neurobehavioural tests as well as functional brain imaging. This study should provide novel evidence for the importance of cortisol rhythmicity in dynamically regulating circuits of the brain responsible for emotional processing, and its potential in the clinical application of glucocorticoid-based therapeutics.

### Trial status

At the time of the manuscript submission (31 August 2015) the trial was ongoing (study status: recruiting).

## Abbreviations

ACTH: corticotrophin (adrenocorticotropic hormone)
ANOVA: analysis of variance
BOLD: blood oxygen level-dependent
CRICBristol: Clinical Research and Imaging Centre (University of Bristol)
ECAT: Emotional Categorisation Task
EMA: ecological momentary assessment
EPST: emotion potentiated startle test
EREC: Emotional Recall Task
ETB: emotional test battery
FDOT: Faces Dot Probe Task
FERT: Facial Expression Recognition Task
fMRI: functional magnetic resonance imaging
HABS: human automated blood sampler
HPA: hypothalamic-pituitary-adrenal (axis)
ICH GCP: international conference for harmonisation of good clinical practice
LSEQ: Leeds Sleep Evaluation Questionnaire
MHRA: Medicines and Healthcare products Regulatory Authority
MRI: magnetic resonance imaging
PIS: participant information sheet
R&D: research and development
REC: research ethics committee
ROIs: regions of interest
UHB: University Hospitals Bristol
